# Genetics and primary care: where are we headed?

**DOI:** 10.1186/s12967-014-0238-6

**Published:** 2014-08-28

**Authors:** Vasiliki Rahimzadeh, Gillian Bartlett

**Affiliations:** Department of Family Medicine, McGill University, 5858 Côte-des-Neiges, Suite 300, Montréal, QC H3S 1Z1 Canada

**Keywords:** Personalized medicine, Ethics, Primary care, Translation, Genomics

## Abstract

Since first sequencing the human genome in 2003, emerging genetic/genomic technologies have ushered in a revolutionary era of medicine that purports to bridge molecular biology and clinical care. The field of translational medicine is charged with mediating this revolution. Sequencing innovations are far outpacing guidelines intended to ease their practice-based applications, including in primary care. As a result, genomic medicine’s full integration in primary care settings especially, has been slow to materialize. Researchers and clinicians alike face substantial challenges in navigating contentious ethical issues raised in translation and implementation, namely preserving the spirit of whole-person approaches to care; maintaining respect for persons and communities; and translating genetic risk into clinical actionability. This commentary therefore explores practical barriers to, and ethical implications of, incorporating genomic technologies in the primary care sector. These ethical challenges are both philosophical and infrastructural. From a primary care perspective, the commentary further reviews the ethical, legal and social implications of the Center for Disease Control’s proposed model for assessing the validity and utility of genomic testing and family health history applications. Lastly, the authors provide recommendations for future translational initiatives that aim to maximize the capacities of genomic medicine, without compromising primary care philosophies and foundations of practice.

“Whatever the ultimate fate of healthcare reform in the US, whatever the limitations of genome-wide association studies, whatever loopholes exist in the Genetic Information Non-discrimination Act of 2008, whatever objections the medical establishment offers to the concept of the masses obtaining their genomic information, make no mistake: the genomes are coming” [1].

## Background

Since the early stages of the Human Genome Project, advances in high throughput sequencing have steered the genomic revolution [2]. Once considered major bottlenecks to expanding sequencing technology outside the research sphere, advances in computational power [3,4], database storage [5] and plummeting costs of whole genome and exome sequencing [6] have made genome medicine a conceivable clinical reality in a matter of years. Genomic medicine, one aspect of personalized medicine, “is a way to customize medical care to your body’s unique genetic makeup” [7]. It enables the development and utilization of novel clinical tools, including next generation sequencing (NGS), to shed light on the complexities of human pathology [8,9]. Advances in genomics demonstrate immense promise in early prevention, characterization and prognostication for “many Mendelian diseases for sure, but potentially for chronic diseases as well” [10]. While current scientific limitations prevent robust sequencing diagnostics for most complex chronic conditions (CCCs), the growing number of genomic/family health history applications for cancer and some CCCs attests to progress in understanding the genetic architecture associated with conditions that are of special interest in primary care (PC) [11].

In order to begin to chart the future of integrating PC and genomic medicine, it is worth returning the historical bases of PC’s purposes and foundations of care. PC is an essential component to maintaining a high-performing healthcare system [12]. In countries with a strong PC system, patients are more likely to receive equitable clinical care, prevention-focused services, and experience fewer health emergencies [12]. PC’s modern roots in the United States can be traced to the Mills Commission Report of 1966. Approximately a decade later, the Alma Ata Conference sought to define the primary care mission and substantiate its role as an international and national healthcare priority. In its definition, the conference used ‘essential,’ ‘scientifically sound’ and ‘socially acceptable’ to describe primary care delivery, and reinforced the impact of primary care on cultivating self-determination and self-reliance in health over the lifecourse [13]. The progressive rhetoric used at Alma Ata, which promoted primary care as a vehicle for improving health education and strengthening community engagement, is reflected in most contemporary conceptualizations of the field today.

### Merging tradition and innovation

As one of the key actors in the treatment and management of complex, chronic conditions (CCCs), PC providers are uniquely situated to translate the benefits of routine genomic profiling for a number of reasons. The routinization of NGS as a standard of care in the diagnosis and treatment of CCC’s could enhance the preventative approaches that PC espouses by identifying genetic predispositions earlier in life. NGS could also facilitate continuity of care by identifying and tailoring disease management plans to patients’ individual genomes throughout the lifecourse. In addition, large-scale sequencing projects conducted as part of genome-wide associated studies (GWAS) are elucidating important genetic patterns and may aid in identifying targeted therapies.

In addition, PC serves as a critical intermediary between public health bodies and upper tiers of the medical institution [12]. As such, PC providers treat the most patients at one time [14], ^a^ thus creating the largest utilization reach for adopters of genomic medicine as well. As a result of the ‘on-the-ground’ accessibility of PC, it is intimately embedded in communities and populations and better able to mitigate access-related disparities in health [15]. Since PC providers are often members themselves of the communities in which they serve, this influences their ability to practice with community-specific, cultural competency and with an understanding of the values and priorities therein. The longitudinal relationships fostered between PC providers and their patient attest to this [16].

Due to the contentious issues genetic/genomic information can raise—such as patient rights ‘not to know,’ [17] disclosure of adult onset conditions in children [18] and prenatal genetic diagnostics [19] to name a few—the greater sociocultural understanding of place may lead to fewer ethical dilemmas in decision-making between patients and healthcare teams. PC providers also tend to have the role of frontline health educators, able to increase genetic literacy among community members at each rung of the socioeconomic ladder. Given public misunderstanding of genetic determinants of health as demonstrated in recent studies [20,21], the need for education before swift integration of genomic medicine is pressing.

## Ethical considerations for translation

### ACCE model: a translation tool for primary care?

The unique facets that characterize the philosophies and foundations of PC are also the foci of debates concerning the application-based ethics of genomic medicine in the PC clinic. These ethical considerations—though certainly not exhaustive—appear in Table 1 and will be discussed in turn in the following sections. This commentary will use as a guide the ethical, legal and social implications (ELSi) component of the ACCE Model Project. The model—which derives it name from the four criteria that are used as the basis for evaluation: *a*nalytic validity, *c*linical validity, *c*linical utility and related *e*thical, legal and social implications—was designed by Center for Disease Control Office of Public Health Genomics (OPHG) to facilitate policy making around proposed uses of genetic/genomic testing in the clinic [22]. The first publicly accessible framework for such evaluation, the ACCE framework, now guides a number of American and international entities on evaluating the practical applications of emerging genetic tests, and has since inspired a separate initiative dedicated solely to genomic applications [23]. It is based on a 44-question survey, 3 of which comprise the ELSi evaluation (Table 2).

**Table 1:** **Summary of translation considerations for genomic medicine and primary care**

**Variable in translation**	**Primary care**	**Genomic medicine**	**ELSi considerations**
Patient population	Families, communities, entire practices	Single, genetically unique patient	Respect for persons; relational decision making;
Technological capacities	Basic, minimal	Data-intensive sequencing machines	Lack of clinical validity and utility for CCCs; professional responsibilities, patient informed consent, disclosure of information, interpreting actionable genetic risks
Meeting health needs	Whole-person, generalist approaches to care; acuity to physical and psychosocial elements of health and wellbeing	Molecular conception of health and disease	Sociocultural and environmental understanding health; supra-genetic determinants of health
Health information	Electronic health record	Electronic health record	Data-intensive storage platforms needed with controlled access; privacy concerns
Graduate and post‐graduate training	Standardized	Under development	Professional responsibilities; lack of specific expertise
Standards of care	Established by professional medical bodies	Under development	Resource and time constraints; professional capacities; management of incidental findings; rights ‘not to know’
Health education	Frontline health educators for global factors of health and disease	Educators on genomic determinants of disease	Resource and time constraints; misunderstanding of genetic determinants of health

**Table 2:** **Evaluation questions 42-44 of the ACCE model**

**ELSi evaluation**	
…	
42	What is known about stigmatization, discrimination, privacy/confidentiality and personal/family social issues?
43	Are there legal issues regarding consent, ownership of data and/or samples, patents, licensing, proprietary testing, obligation to disclose, or reporting requirements?
44	What safeguards have been described and are these safeguards in place and effective?

### Impact of scientific limitations on risk and decision-making

To date, the lack of analytical validity and clinical utility for many NGS technologies in diagnosing or treating CCCs simply cannot meet the current regulatory evidence standards for clinical implementation—let alone for coverage by major health insurers^b^ [24]. Because a vast majority of PC consultations involve management of CCC, the power of genomic medicine in the PC clinic relies in the ability to alter health behaviors in line with communicated risks from genomic analyses. Despite this, research suggests PC providers frequently lack expertise in extrapolating clinical risk from genomic profiles, and report feeling unprepared to incorporate genomic medicine in routine clinical practice [25,26] due in part to limited expertise and relevant training^c^. Both communication and interpretation of genetic risks are inextricably linked to bioethical principles of informed consent and beneficence, among others. If patients were to base clinical decisions on results from such tests, to provide (uncertain) risk of predisposition would be negligent at best.

### Personalization and respect for persons

Though the ELSi evaluation of the ACCE model offers a useful guide for identifying pressing concerns, some are calling the absence of a ‘personal utility’ measure a major limitation: “Clinical utility conceived only as improved morbidity and mortality fails to appreciate personal utility for the patient, such as the financial and psychological benefits of resolution of an unknown diagnosis, changes in life-style leading to overall improvements in health, or even family planning, all of which are influenced by knowledge of the genetic susceptibility of disease” [24]. The contextual and sociocultural focus of a proposed ‘personal utility’ measure is necessary if PC providers are to fulfill the bioethical mandate of respect for persons. A personal utility measure would also allow PC providers to better assess the true value of genomic applications in the local milieu.

### Health needs beyond the genome

Other challenges in the translation process require critical analysis of how genomic medicine problematizes some of the foundations of the primary care philosophy, specifically its commitment to whole-person approaches. Some claim genomic technologies may even run counter to the whole- person care approaches often used in PC [27], and in pursuit of advanced care in the 21^st^ century that conforms to “care we need and no less, the care we want and no more” [28]. Though whole person care cannot be precisely defined—inasmuch as it reflects a professional practice of connecting with, and recognizing the complexity of, patients in their pursuit of health and wellbeing—whole person care transcends ‘curing’ in a clinical encounter to incorporate a more humanistic and patient-centered approach towards ‘healing’ [29]^d^.

### Behavior modification

PC providers’ focus on the myriad influences on health over the lifecourse, as well as tackling determinants of ill-health, requires a longitudinal review of the genome that may change in response to adverse life events, environmental factors and/or specific health behaviors. As a result, clinical care based on discrete analyses of the genome in moments of illness or health threatens to ignore the changes that DNA can incur over time [30]. Furthermore, genomic medicine may not always offer the appropriate clinical tools to address all patient needs. With increasing attention and funding dedicated to translating the benefits of NGS, there is nevertheless a critical role for whole person care and its focus on the supra-genetic components of physical, mental and spiritual health: “In viewing the patient as a hub in a wheel of a complex social, psychological and physical environment, we must attend to the impact of a diagnosis and focus our energies on maximizing the patient’s adjustment and coping” [31]. In this way, PC providers often address patient needs for which the genome may not provide information on effective therapies, such as emotional or mental health support [32,33].

Some argue genetic reductionism—generally an overreliance on genetic information to predict health outcomes—is an affront to the culture of caring itself [34]. Studies on lay knowledge of genetic determinants of health report that many patients rationalize noncompliance in health management plans due to a belief in the deterministic nature of their genetics [35-37]. Because primary care medicine is predicated on the idea that care is delivered through addressing an array of health needs over the lifecourse [38], this philosophy can challenge the strictly molecular conceptions of disease that is often characteristic of genomic approaches.

### Duty to the community

The clinical reach of primary care providers often encompasses communities of varying sizes and structures. The *micro*-communities of family units present valuable insight into the needs of the *macro*-community, which may comprise larger urban cities and towns in which families congregate. Ideally, PC strives to bring healthcare closest to where people live and work, while addressing the prevailing concerns associated with each in a culturally relevant manner [39-41]. Therefore PC providers become critical intermediaries between the social structures of place, and its impact on the health profiles of populations within a given community. The responsibility for health outcomes within communities of people throughout the lifecourse challenges the strictly precision care that genomic medicine promotes. Primary care providers are often charged with practicing medicine through a population health lens as well [12], an aspect of the profession that could run counter to the strictly individualist approach that genomic medicine espouses. Translation should therefore aim to preserve and strengthen, as opposed to supplant population-based benefits of primary care services: “Systems that integrate care both horizontally for individuals, communities, and populations and vertically for specific diseases are most likely to provide the greatest value. Currently, vertical integration of care for disease is rewarded and supported to a greater degree than horizontal integration of care for people and populations” [42].

### Justice: resource allocation and health inequities

The heterogeneity of the PC clinic can pose operational difficulties and resource limitations for genomic-medicine translation initiatives [43]. Kerner et al. concur that, “most translational research is conducted in the relatively resource-rich infrastructures of either academic medical centers or the biomedical industry” [44]. The authors argue current translational research may not necessarily reveal how related technology will perform in an under-resourced setting, such as free community clinics. More concerning, however, is the “new association of genomic medicine with ‘precision medicine.’ It reinforces the notion that absent genomic apparatuses, clinicians who deliver healthcare are imprecise at best, and dangerous at worst” [45,46].

Although the Affordable Care Act (ACA) attempts to remedy such health disparities through insurance reforms applicable to all healthcare tiers, primary care expansion will continue to be the driving force towards realizing a more equitable healthcare system. Thus, in order to envision a primary care future where genomic medicine is a component, researchers and practitioners alike must take into account the influx of new patients that will now be afforded access to this basic level of care. That is, genomic medicine must be available and operational at the most under-resourced and diverse primary care settings if it hopes to prevent widening the already significant health disparities witnessed in the U.S^e^.

### Data storage and privacy concerns

Infrastructural demands, such as establishing secure information-sharing platforms, have also emerged in light of the looming clinical translation [47,48]. The gatekeeping function of PC means providers must efficiently distribute patient health information to appropriate specialists as needed. The distribution of electronic health records that also patients’ genomic profiles will necessarily be data-intensive, and require sophisticated access-control mechanisms to meet Health Insurance Portability and Accountability Act (HIPAA) regulations.

### Educational pre-requisites

Public opinion indicating gross misunderstanding about the basic science of genomic medicine necessitates educational initiatives that need to keep pace with integration efforts. The absence of such initiatives threatens to undermine the most fundamental bioethical rights, such as informed consent, among patients with low genetic and health literacy. Enabling patients to act as partners in managing their own health involves being on the frontlines of health education, an increasingly complicated effort when (questionable) medical information is easily accessed online. More importantly, educating patients presumes that primary care providers themselves are well versed in understanding genetic medicine in order to make meaningful diagnoses, an assumption that is far from current medical education reality according to recent surveys [49].

In responding to patient inquiries about these emerging innovations in medical practice, “there is substantial reason to believe that the health care providers fielding such questions will be primary care providers” [50]. Despite potentially overwhelming the PC system with an influx of patients and with low genetic literacy, some warn against the dangers of allowing curious patients to seek clinical guidance from entities outside the formal clinical institution, such as from direct-to-consumer entities [51,52].

### What to do about incidental findings?

No issue illustrates the irony of “personalized” genomics in PC better than the disclosure of incidental findings. By nature, genetic information is both shared and identifying [53]. With future standards of care based on whole genome/exome sequencing, incidental findings will cease to become ‘incidental,’ and non-disclosure grounds for legal action^f^. In the context of PC, the ethical and legal obligations to disclose findings from genomic analyses are complicated by virtue of treating multiple patients within the same micro community. The discovery of a clinically significant incidental finding that is actionable yet implicates multiple members of a biologically related family, typifies the ethical dilemmas PC physicians may face in determining unto who their duty to inform rests. Such situations can contribute to “personal/family social issues” (Table 2) when care providers must manage confidentiality obligations with multiple patients within a family^g^. In some cases, inability to interpret actual clinical risk from genetic/genomic testing may prevent necessary healthcare intervention (s) either immediately or in the future. This issue is exacerbated in the absence of standards of care for many CCCs that do not yet routinize genetic or genomic diagnostic tools.

## Conclusion

The primary care arena presents unique challenges to the evaluation, diffusion and implementation of genomic technologies [54]. The clinical landscape is in both scientific and legislative transition, generating public discourse on the meanings of health and healthcare. Yet the same aspects that present limitations also render how the primary care setting is a critical forum in which to translate the benefits of personalized medicine in practice. Evaluating the quality of healthcare services is to examine, among others, the purpose and avenues for their delivery. Where genomics delivers molecular understanding of determinants underlying diseases, good primary care aims to situate this information against the sociocultural backdrop in which patients live. In this way, PC strives to appreciate the ‘whole person’ dimensions of an individual’s health status. Therefore the authors propose the F^3^ approach, a triangulated strategy to i) **F**ocus on the local context, ii) **F**orge partnerships and support iii) **F**urther research. In actualizing the F^3^ strategy, genomic applications in the primary care setting facilitates needs-assessments and priority setting in diverse primary care venues. Forging partnerships between PC providers and additional health professionals including genetic counselors as well as genetic researchers, will be valuable in coordinating and designing implementation strategies for PC; because of PC’s embeddedness within communities, promoting and supporting translation research that engages stakeholder communities on perceptions of genomic medicine is essential.

To a greater degree than most specialties, primary care is charged with health education, advocacy and prevention. As such, implementing genomic medicine tools should facilitate the preservation of the whole person care philosophy, fulfill basic principles of medical ethics, and develop tools for translating genetic risk of common chronic disease into clinical actionability. This process must be strategic and specific, using translation frameworks applicable to the unique challenges of PC if it is to maximize benefits of genomic and personalized medicine in PC.

## Endnotes

^a^Americans made 1 billion visits to the doctor in 2008. Nearly 50% of visits (462 million) were to primary care doctors [14].

^b^There have been initiatives, however, to systematically catalogue genetic variants based on their clinical actionability [55], and to establish which genomic testing and/or family history applications in fact have demonstrated sufficient evidence to warrant their implementation at the bedside [56].

^c^In one study assessing familiarity with genomic testing, over 49 percent of primary care physicians admitted their clinical training was not adequate to perform such testing regularly [49].

^d^Hutchinson writes, “Curing is an action carried out by the health care practitioner to eradicate disease or correct a problem, while healing is a process leading to a greater sense of integrity and wholeness …The roles of the patient and of the health care practitioner in curing versus healing are not just different, they are diametrically opposed” [29].

^e^Genomic medicine researcher and family physician, Gregory Feero, shares this concern: “Personalized genomics in its current form represents a potentially disruptive technology that will both empower and imperil consumers who are early adopters. Depending on the level of public uptake of these services, the downstream consequences could be profound. At a minimum, the wash of data will stress the capacity of the current model for the provision of genetic services and place pressure on primary care practitioners to react to the informational barrage in a vacuum of solid evidence-based guidelines. Given the overall health care environment, mismanagement of this type of genomic data could worsen the fiscal crisis and widen health care disparities” [50].

^f^A recent study published by author VR testifies to the professional complexities of disclosing incidental findings in the context of pediatric research. Health professionals and clinical researchers report disclosure decisions should be context specific, relying on such information as the clinical significance of the finding, respect for individual, scope of professional responsibilities, and implications for the healthcare/research system [57].

^g^Complexities in familial disclosure of incidental findings have led to civil litigation in numerous states. One such case, *Kimberly A. Molloy v. Diane M. Meier MD (2004),* the Minnesota Supreme Court ruled that a “physician’s duty regarding genetic testing and diagnosis extended beyond the patient to biological family members who might be harmed” [58]. Numerous wrongful birth lawsuits filed in the United States reflect the uncertainty inherent to incidental finding disclosure policies. The Ohio Supreme Court recognized a wrongful birth claim for the first time in *Schirmer V. Mt. Auburn Obstetrics & Gynecologic Associates Inc. (*2006). In this case, the parents were permitted to sue their physician for the (wrongful) birth of their disabled child after receiving negligent medical advice from physicians following genetic test results [58].

## Abbreviations

ACCE: Analytic validity, Clinical validity, Clinical utility and Ethical, legal and social considerations
CCC: Complex, chronic conditions
NGS: Next generation sequencing
PC: Primary care
